# Associations between anxiety, depression with migraine, and migraine-related burdens

**DOI:** 10.3389/fneur.2023.1090878

**Published:** 2023-04-25

**Authors:** Shaojie Duan, Zhiying Ren, Hui Xia, Ziyao Wang, Tao Zheng, Guanglu Li, Lei Liu, Zunjing Liu

**Affiliations:** ^1^Department of Geriatrics, Taizhou Central Hospital (Taizhou University Hospital), Taizhou, China; ^2^Graduate School of Beijing University of Chinese Medicine, Beijing, China; ^3^Department of Neurology, China-Japan Friendship Hospital, Beijing, China; ^4^Department of Neurology, Peking University People’s Hospital, Beijing, China

**Keywords:** anxiety, depression, migraine, migraine burden, association

## Abstract

**Background:**

Anxiety and depression are the most common psychiatric comorbidities in migraine, but their impact on the risk of developing migraine and their gender and age differences are unclear, and research on their associations with migraine-related burdens are limited.

**Objective:**

To systematically explore the association between anxiety and depression with migraine and migraine-related burdens, including the risk of developing migraine, as well as migraine frequency, severity, disability, headache impact, quality of life and sleep quality.

**Methods:**

A total of 170 migraineurs and 85 sex-and age-matched healthy control subjects were recruited consecutively for this study. Anxiety and depression were assessed using Zung’s Self-rating Anxiety Scale (SAS) and Self-rating Depression Scale (SDS), respectively. Logistic regression and linear regression analyses were used to explore the associations between anxiety and depression with migraine and its burdens. The receiver operating characteristic (ROC) curve was used to evaluate the predictive value of SAS score and SDS score on migraine and its severe burdens.

**Results:**

After adjusting for confounders, anxiety and depression remained significantly associated with an increased risk of developing migraine, with odds ratios of 5.186 (95% CI:1.755–15.322) and 3.147 (95% CI:1.387–7.141), respectively. Meanwhile, there were significant additive interactions between the association of anxiety and depression with the risk of developing migraine in gender and age (*P* for interaction <0.05), and the stronger correlations were found in participants with an age ≤ 36 years old and females. In addition, anxiety and depression were significantly independently associated with the migraine frequency, severity, disability, headache impact, quality of life, and sleep quality in migraine patients (*P* trend <0.05). The area under the ROC curve (AUC) of SAS score in predicting developing migraine was significantly higher than that of SDS score [0.749 (95% CI: 0.691–0.801) vs. 0.633 (95% CI: 0.571–0.692), *p* < 0.0001].

**Conclusion:**

Anxiety and depression were significantly independently associated with the increased risk of migraine and migraine-related burdens. Enhanced assessment of SAS score and SDS score is of great clinical value for the early prevention and treatment of migraine and its burdens.

## Introduction

Migraine, as one of the most common neurological disorders characterized by multiphase attacks of head pain and reversible neurological and systemic symptoms (1, 2), is the second most disabling neurological disorder and the third most prevalent medical condition in the world (3, 4). The annual and lifetime prevalence was 6 and 13% in men and 18 and 33% in women (1). Prior to puberty, boys and girls are equally affected, but the female preponderance emerges after puberty, and the prevalence peaks among the ages of 35 and 39 years (5). Migraine can increase the risk of cerebrovascular disease and reduce the health-related quality of life and has a substantial effect on daily activities and direct medical costs (1, 6, 7). Given the severe consequences of migraine-related burden on families and society, this condition is a common concern for many patients and their physicians (8–10). Notably, there are also stark gender differences in migraine-related burdens. Men with migraine generally have less severe attacks and disabilities and are less likely to receive a diagnosis than women with migraine (11). Despite the understanding of migraine pathophysiology and the evidence-based guidelines intended to inform clinical decision-making with migraine are advancing rapidly, the prevention and treatment of migraine remains suboptimal (1, 12).

Migraine is known to be associated with a variety of psychiatric disorders, and the bidirectional relationship between psychiatric disorders and migraine has received more and more research and attention (7). Anxiety, depression, stress, post-traumatic stress disorder, and bipolar disorder are common psychiatric disorders associated with migraine, of which depression and anxiety are the most common (7, 13). Migraine patients are more likely to suffer from anxiety and depression compared with people without migraine, and anxiety and depression can also increase the risk of migraine (7, 14–17). However, given gender and age differences in migraine, it is unclear whether the effects of anxiety and depression on migraine risk differ by age and gender.

Our previous study showed that poor sleep quality was significantly and independently associated with an increased risk of developing migraine and migraine-related burdens (18). Anxiety and depression, as mental diseases closely related to sleep quality, their association with migraine and migraine-related burdens also need further research. Previous studies confirmed that migraine combined with anxiety and depression can increase the risk of migraine chronicity, poor treatment outcomes, and medical costs (19), and the higher frequency of migraines was associated with higher scores for anxiety and depressive symptoms (20). In addition, previous studies have reported that depression, anxiety, and emotion regulation difficulties in patients with medication overuse headache may have a negative impact on their headache (21). Emotional regulation associated with pain and psychological complications may be an important risk factor for the development and maintenance of chronic pain (22). However, the relationship between anxiety and depression with migraine attack frequency, severity, disability, and quality of life in migraineurs are unclear, and the gender and age differences need to be further investigated.

Zung’s Self-Assessment Scale for Anxiety (SAS) and Depression Self-Rating Scale (SDS) are commonly used clinical scales for anxiety and depression assessment (23), and their reliability and validity have been validated in Chinese populations (24, 25). Considering the close relationship between anxiety and depression and migraine and its burden, SAS and SDS score may be potential predictors of developing migraine or progression of migraine burden. However, few studies have reported the predictive value of the SAS and SDS score for migraine.

Therefore, the main objectives of this study are as follows: (1) To investigate the effect of anxiety and depression on the risk of developing migraine, and to analyze the gender and age differences between them. (2) To investigate the relationship between anxiety and depression with the migraine frequency, severity, disability, quality of life, and sleep quality of migraine patients. (3) To initially assessed the predictive value of the SAS and SDS score on migraine and its burdens, aiming to provide new ideas for early prevention and treatment of migraine.

## Materials and methods

### Study design

The design of this study mainly consists of two parts. For the specific study protocol, please refer to our previous study.^1^ This study expanded the sample size of subjects from the previous one. First, a case–control study method was used to match 170 migraine patients and 85 healthy control subjects according to gender and age to explore the effect of anxiety and depression on the risk of developing migraine. Then, these 170 migraine patients were further analyzed to explore the relationships between anxiety and depression comorbidities in migraine patients and migraine frequency, severity, disability, and related life and sleep burden of migraine.

### Study participants

According to the International Classification of Headache Disorders 3rd edition (ICHD-III) diagnostic criteria for migraine, we consecutively included 170 migraine subjects (including 20 with aura and 150 without aura migraine) who visited the Beijing China-Japan Friendship Hospital Neurology Headache Clinic between February 2021 and September 2022, including 145 females and 25 males, with an average age of 37.17 ± 8.47 years old. They were all paroxysmal migraines, with an average monthly attack frequency of 5.045 ± 5.23 times in the past month, at least once a month and at most 30 times.

At the same time, we also included 85 gender- and age-matched healthy control volunteers without migraine, including 71 females and 14 males, with an average age of 35.05 ± 8.41 years old. All healthy control volunteers were required to have no family history of migraine and no current or previous diagnosis of primary or secondary migraine. All subjects underwent professional diagnostic assessments and completed standardized questionnaires interviewed by certified neurologists and headache specialists. All subjects voluntarily participated in this study.

### Anxiety and depression assessment

In this study, anxiety and depression were assessed using Zung’s Self-rating Anxiety Scale (SAS) and Self-rating Depression Scale (SDS), respectively (23), whose reliability and validity have been validated in the Chinese population (24, 25). Both the two assessment scales include 20 items, with raw scale scores for range from 20 to 80 and the transformed index score ranging from 25 to 100, a higher score indicates a higher level of anxiety and depression. Based on previous studies using SAS and SDS scores to assess anxiety and depression in Chinese populations, subjects with SAS total transformed score ≧50 were classified as anxiety, and subjects with SDS total transformed score ≧53 were classified as depression (26, 27).

### Visual analog scale pain score

The visual analog scale (VAS) pain score is one of the most widely used to assess the subjective pain intensity of patients with migraine (28, 29). The pain intensity was determined by asking, “On a scale of 0 to 10 (where 0 = no pain at all and 10 = pain to the extreme), on average how painful are your headaches?”

### Migraine disability assessment questionnaire

The Migraine Disability Assessment (MIDAS) is a 5-item questionnaire used to quantify migraine-related disability (30), which has been proven in previous studies to have high reliability and high internal consistency (31, 32). A higher cumulative total score indicates a higher degree of disability in migraineurs. When the total score is >21, it is defined as a severe disability. (33).

### Headache impact test

The Headache Impact Test (HIT-6) is a self-report questionnaire designed to assess the impact of headaches on quality of life (34), and its validity and reliability have been validated in patients with various types of headaches, including primary and secondary headaches (35), episodic and chronic migraine (36–38). The questionnaire consists of 6 items that cumulative total score ranges from 36 to 78 points. The higher the score, the greater the impact of the headache on the patient’s life. Severe headache impact is defined when the total score is ≥60 (39).

### Migraine-specific quality-of-life questionnaire

The Migraine-Specific Quality of Life Scale (MSQ) (Version 2.1) is one of the most widely used measures of the impact of migraine on quality of life that consists of three subscales, including role function-restrictive, role function-preventive, and emotional function (40). In this study, we used the Migraine-Specific Quality of Life Questionnaire Chinese version 2.1 (MSQv2.1-C), which exhibited satisfactory reliability and validity in a sample of individuals with migraine who speak Chinese (41). A higher cumulative total score indicates better quality of life for migraine patients.

### Pittsburgh sleep quality index

The Pittsburgh Sleep Quality Index (PSQI) is a common self-rated questionnaire to assess sleep quality (42). It included 19 items in seven components, including subjective sleep quality, sleep latency, sleep time, habitual sleep efficiency, sleep disorders, sleep medication, and daytime dysfunction (43). A higher cumulative total score indicates worse sleep quality, and the poor sleep quality is defined when PSQI score is >5 (44).

### Other assessments

In addition, through standardized questionnaire interviews and quality control, the demographic and baseline data characteristics such as age, gender, height, weight, smoking history, drinking history, exercise time, and subjective pressure were collected. Body mass index (BMI) was calculated as the weight (in kilograms) divided by the square of the height (in meters). The definition of smoking history and drinking history referred to the current or previous behavior of smoking and drinking. Weekly exercise time referred to the total exercise time (hour) per week. Subjective pressure was evaluated by a visual analog scale pressure score, with a range of zero to ten.

### Statistical analysis

First, baseline characteristics of the migraine and control groups were compared. The independent samples *t*-test was used to compare normally distributed quantitative data between groups and expressed as mean and standard deviation (mean ± SD). The Mann–Whitney *U* test was used to compare non-normally distributed quantitative data between groups, expressed as median and interquartile ranges [M (P25, P75)]. The chi-square test was used to compare categorical data between groups, expressed as number of cases and percentage [n (%)]. Then, logistic regression analysis was conducted to evaluate the effect of anxiety and depression on the risk of migraine under different adjustment conditions and different subgroups, and to explore the interaction of anxiety and depression with other confounders on the risk of migraine. Furthermore, for migraine subjects, multiple linear regression analysis were further performed to evaluate the associations of anxiety and depression with migraine attack frequency, VAS score, MIDAS score, HIT-6 score, MSQ score and PSQI score. Multivariate logistic regression was used to analyze the effect of anxiety and depression on poor sleep quality, severe disability degree, and severe headache impact in migraine subjects. In addition, the receiver operating characteristic (ROC) curves were used to assess the predictive value of SAS and SDS scores on migraine and the severe burden of migraine.

All statistical tests were two-tailed and were considered significant for *P* less than 0.05 (*p*-values <0.05). Statistical analyses were performed using Statistical Package for the Sciences (SPSS, version 25.0) and MedCalc statistical software (version 19.6.4).

## Results

### Baseline characteristics of participants

The baseline characteristics of migraine and control groups were presented in Table 1. There was no significant difference in age, gender, BMI, smoking history, drinking history, exercise, and pressure score between migraine patients and healthy control subjects (all *p*-values >0.05). Migraine patients had significantly higher SAS and SDS scores than healthy control subjects (all *p*-values <0.05). There were 22.4% of migraine patients with anxiety and 25.9% of migraine patients with depression, which was significantly higher than the incidence of anxiety (4.7%) and depression (9.4%) in healthy control subjects (all *p*-values <0.05).

**Table 1:** Baseline characteristics of migraine and control groups.

Variable	Total (*N* = 255)	Control (*N* = 85)	Migraine (*N* = 170)	*p*-value^*^
Gender [n (%)]				0.715
Male	39 (15.3%)	14 (16.4%)	25 (14.7%)	
Female	216 (84.7%)	71 (83.5%)	145 (85.3%)	
Age, years (mean ± SD)	36.46 ± 8.49	35.05 ± 8.41	37.17 ± 8.47	0.060
BMI, kg/m2 (mean ± SD)	22.07 ± 3.57	22.20 ± 3.74	22.01 ± 3.49	0.691
Smoking history [n (%)]	26 (10.2%)	5 (5.9%)	21 (12.4%)	0.127
Drinking history [n (%)]	52 (20.4%)	17 (20.0%)	35 (20.6%)	1.000
Weekly exercise time, hours (mean ± SD)	0.97 ± 1.96	0.93 ± 2.04	1.00 ± 1.93	0.791
Pressure score (mean ± SD)	5.10 ± 2.48	5.36 ± 2.46	4.97 ± 2.48	0.251
SAS score [M (P25, P75)]	38.8 (33.8,46.3)	33.8 (30.0,38.8)	42.5 (35.0,48.8)	<0.001
Anxiety [n (%)]	42 (16.5%)	4 (4.7%)	38 (22.4%)	<0.001
SDS score [M (P25, P75)]	38.8 (33.8,50.0)	36.3 (31.3,42.5)	40.0 (34.7,52.5)	0.001
Depression [n (%)]	52 (20.4%)	8 (9.4%)	44 (25.9%)	0.002

BMI, body mass index; SAS, Self-rating Anxiety Scale; SDS, Self-rating Depression Scale.^*^Categorical variables were calculated by chi-square test, and Fisher’s exact test was used when the sample size was less than five. Quantitative variables were calculated by the Mann–Whitney U test or independent samples *t*-test.

### Effect of anxiety and depression on the risk of developing migraine

Multivariate logistic regression analyses were conducted to explore the effect of anxiety and depression on the risk of developing migraine. It can be seen from Table 2 that the SAS score, SDS score, anxiety, and depression were significantly positively correlated with the risk of migraine (all *p*-values <0.05). After adjusting for age, gender, smoking history, drinking history, BMI, weekly exercise time, and pressure score, the odds ratios (ORs) for a 1-SD increase in SAS score and SDS score were still 1.118 (95%CI:1.074–1.164) and 1.047 (95%CI:1.017–1.077), respectively. Compared with subjects without anxiety and depression, subjects with anxiety and depression had a significantly increased risk of migraine, with OR values of 5.186 (95%CI:1.755–15.322) and 3.147 (95%CI:1.387–7.141), respectively.

**Table 2:** Multivariate logistic regression analysis of anxiety and depression on migraine.

Variable		β	SE	Wald *χ*^2^	*p*-value	OR (95%CI)
Model 1	SAS score	0.118	0.02	34.133	<0.001	1.125 (1.081–1.170)
	Anxiety	1.763	0.544	10.491	0.001	5.830 (2.006–16.94)
	SDS score	0.048	0.014	11.648	0.001	1.049 (1.020–1.078)
	Depression	1.212	0.411	8.714	0.003	3.361 (1.503–7.517)
Model 2	SAS score	0.118	0.02	34.133	<0.001	1.125 (1.081–1.170)
	Anxiety	1.831	0.547	11.194	0.001	6.242 (2.135–18.248)
	SDS score	0.048	0.014	11.959	0.001	1.050 (1.021–1.079)
	Depression	1.251	0.414	9.148	0.002	3.495 (1.553–7.864)
Model 3	SAS score	0.112	0.02	29.679	<0.001	1.118 (1.074–1.164)
	Anxiety	1.646	0.553	8.868	0.003	5.186 (1.755–15.322)
	SDS score	0.046	0.014	9.943	0.002	1.047 (1.017–1.077)
	Depression	1.146	0.418	7.517	0.006	3.147 (1.387–7.141)

SAS, Self-rating Anxiety Scale; SDS, Self-rating Depression Scale. Model 1: Unadjusted. Model 2: Adjusted for age and gender. Model 3: Adjusted for age, gender, smoking history, drinking history, BMI, weekly exercise time, and pressure score.

### Effect of anxiety and depression on the risk of migraine stratified by subgroups

To further investigate the impact of other confounding factors on the correlation between anxiety, depression with the risk of developing migraine, subgroup analyses were carried out according to gender, age, smoking history, and drinking history. Table 3 summarized the results of the subgroup analysis and the interaction results. After adjusting for age, gender, smoking history, drinking history, BMI, weekly exercise time, and pressure score, there were still significant additive interactions between anxiety and depression with the risk of developing migraine in gender, age (*P* for interaction <0.05), and the stronger correlations were found in participants with an age ≤ 36 years old (median age) and females.

**Table 3:** Effect of anxiety and depression on migraine stratified by subgroups.

Subgroups	Anxiety			Depression		
	OR (95%CI)	*p*-value	*p* for interaction^*^	OR (95%CI)	*p*-value	*p* for interaction^*^
Age			0.005			0.024
≥36 y	3.669 (0.765–17.593)	0.104		1.591 (0.517–4.895)	0.418	
<36 y	7.926 (1.587–39.579)	0.012		5.682 (1.459–22.130)	0.012	
Gender			0.007			0.034
Males	7.664 (0.606–96.946)	0.116		——	0.999	
Females	6.293 (1.779–22.262)	0.004		2.765 (1.168–6.545)	0.021	
Smoking history			0.999			0.731
Yes	——	0.995		——	0.997	
No	5.237 (1.716–15.982)	0.004		3.763 (1.465–9.663)	0.006	
Drinking history			0.099			0.221
Yes	10.307 (1.037–102.434)	0.046		5.548 (0.512–60.152)	0.159	
No	5.870 (1.590–21.666)	0.008		3.347 (1.308–8.566)	0.012	

^*^Adjusted for age, gender, smoking history, drinking history, BMI, weekly exercise time, and pressure score.

### Association of anxiety and depression with migraine-related burdens

**Figure 1:**
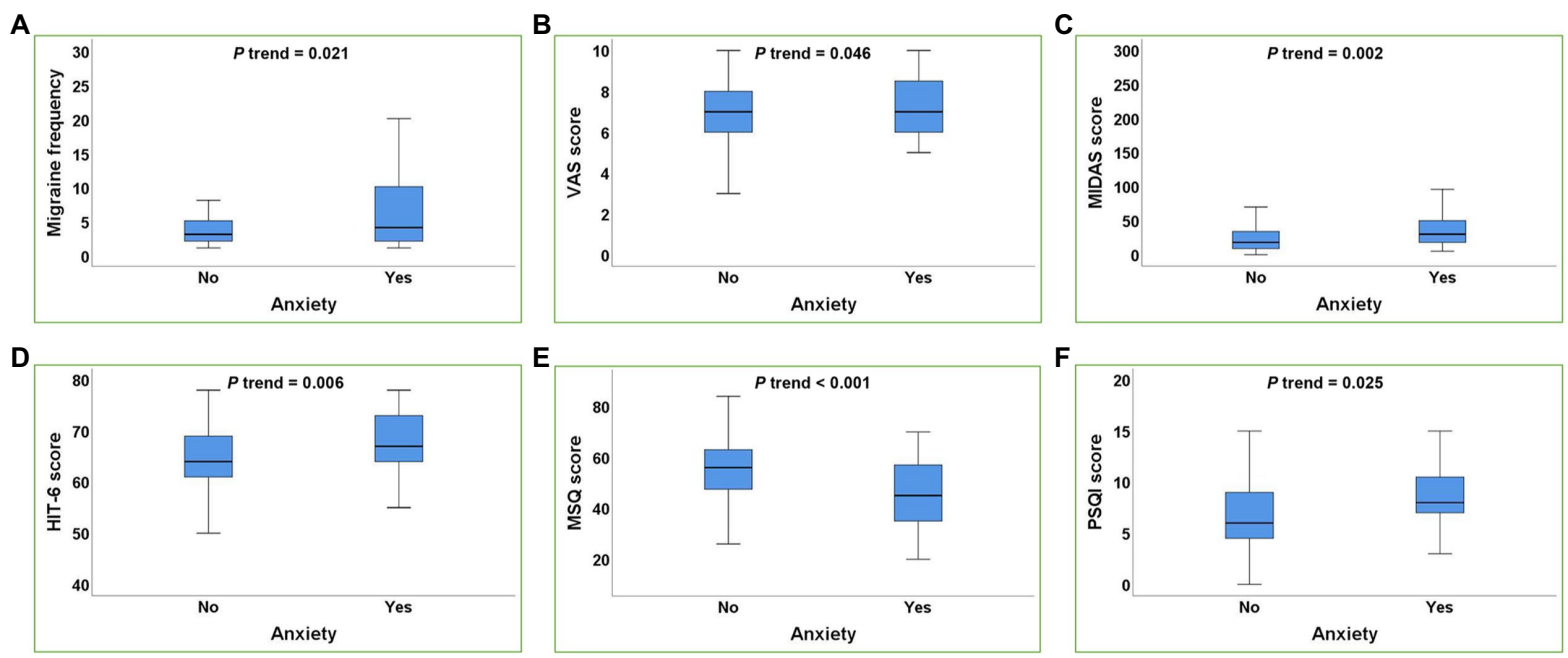
Box plot of the relationship between anxiety and migraine-related burdens. **(A–F)**: Anxiety was positively correlated with the migraine frequency **(A)**, VAS score **(B)**, MIDAS score **(C)**, HIT-6 score **(D)**, PSQI score **(F)** and negatively correlated with MSQ score **(E)** in migraine patients (all *p* trend<0.05). *p* trend value: Adjusted for age, gender, smoking history, drinking history, BMI, weekly exercise time, pressure score, migraine aura.

To further investigate the associations between anxiety and depression with the migraine-related burdens, we divided the migraine subjects into subgroups according to whether or not they combined with anxiety or depression. As shown in Table 4 and Figure 1, the migraine frequency, VAS score, MIDAS score, HIT-6 score, and PSQI score of migraine patients with anxiety were significantly higher than those without anxiety, and the MSQ score was significantly lower than those without anxiety. After adjusting for age, gender, smoking history, drinking history, BMI, weekly exercise time, pressure score, migraine aura, anxiety was still significantly correlated with migraine frequency, VAS score, MIDAS score, HIT-6 score, MSQ score, and PSQI score (all *P* trend <0.05).

**Table 4:** Multivariate linear regression analysis of the effects of anxiety and depression on migraine-related burden.

Variable	Anxiety			Depression		
	Yes (*n* = 38)	NO (*n* = 132)	*p* trend ^*^	Yes (*n* = 44)	NO (*n* = 126)	*p* trend ^*^
Migraine frequency	4.0 (2.0, 10.0)	3.0 (2.0, 5.0)	0.021	4.0 (2.0, 9.5)	3.0 (2.0, 5.0)	0.043
VAS score	7.0 (6.0, 8.6)	7.0 (6.0, 8.0)	0.046	7.0 (7.75, 8.0)	7.0 (6.0, 8.0)	0.030
MIDAS score	30.0 (17.5, 51.3)	18.0 (9.0, 34.0)	0.002	28.25 (18.0, 48.8)	15.0 (9.0, 34.0)	0.024
HIT-6 score	67.0 (64.0, 73.0)	64.0 (61.0, 69.0)	0.006	66.0 (63.0, 72.0)	64.5 (61.0, 69.3)	0.004
MSQ score	45.0 (35.0, 57.0)	56.0 (47.3, 63.0)	<0.001	50.0 (39.0, 57.0)	56.0 (45.0, 64.3)	0.002
PSQI score	8.0 (6.8, 10.9)	6.0 (4.3, 9.0)	0.025	8.0 (7.0, 10.9)	6.0 (4.0, 8.3)	0.001

VAS, Visual analog scale; HIT-6, Headache Impact Test; MIDAS, Migraine Disability Assessment; MSQ, Migraine-Specific Quality-of-Life Questionnaire; PSQI, Pittsburgh Sleep Quality Index.

^*^Adjusted for age, gender, smoking history, drinking history, BMI, weekly exercise time, pressure score, migraine aura.

**Figure 2:**
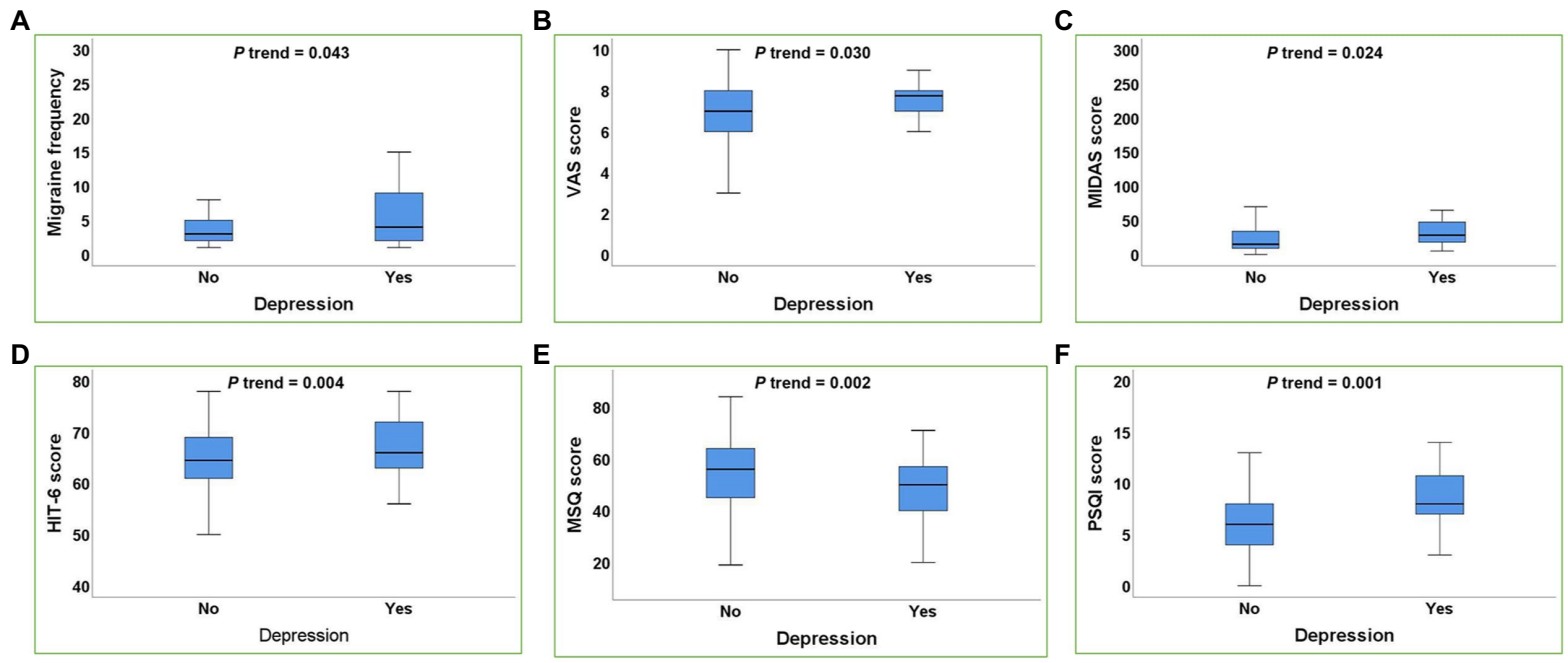
Box plot of the relationship between depression and migraine-related burdens. **(A–F)**: Depression was positively correlated with the migraine frequency **(A)**, VAS score **(B)**, MIDAS score **(C)**, HIT-6 score **(D)**, PSQI score **(F)** and negatively correlated with MSQ score **(E)** in migraine patients (all *p* trend<0.05). *p* trend value: Adjusted for age, gender, smoking history, drinking history, BMI, weekly exercise time, pressure score, migraine aura.

Similar results can be seen in depression, after adjusting for confounding factors, depression was significantly correlated with the migraine frequency, VAS score, MIDAS score, HIT-6 score, MSQ score, and PSQI score (all *p* trend <0.05) (Figure 2).

### Effects of anxiety and depression on poor sleep quality, severe disability degree, and severe headache impact in migraine patients

To further explore the effect of anxiety and depression on the severe burden of migraine in migraine patients, we used poor sleep quality, severe disability degree, and severe headache impact as dependent variables, respectively, to explore the effect of anxiety and depression on their risk of occurrence. As shown in Table 5, after adjusting for age, gender, smoking history, drinking history, BMI, weekly exercise time, pressure score and migraine aura, SAS score, SDS score, anxiety and depression were significantly positively correlated with the risk of poor sleep quality, severe disability degree, and severe headache impact (all *p*-values <0.05). The risk of poor sleep quality, severe headache impact, and severe disability degree in migraineurs with anxiety were 3.120, 5.881, and 3.587 times than that in migraineurs without anxiety, respectively. The risk of poor sleep quality, severe headache impact, and severe disability degree in migraineurs with depression were 4.913, 26.968, and 3.481 times than that in migraineurs without anxiety, respectively (all *p*-values <0.05).

**Table 5:** Effects of anxiety and depression on poor sleep quality, severe disability degree, and severe headache impact in migraine patients.

Variable	Poor sleep quality		Severe headache impact		Severe disability degree	
	OR (95%CI)	*p*-value^*^	OR (95%CI)	*p*-value^*^	OR (95%CI)	*p*-value^*^
SAS score	1.085 (1.037–1.135)	<0.001	1.119 (1.052–1.192)	<0.001	1.083 (1.038–1.130)	<0.001
Anxiety	3.120 (1.137–8.556)	0.027	5.881 (1.140–30.330)	0.034	3.587 (1.481–8.684)	0.005
SDS score	1.074 (1.033–1.117)	<0.001	1.129 (1.061–1.202)	<0.001	1.045 (1.013–1.078)	0.006
Depression	4.913 (1.746–13.824)	0.003	26.968 (2.664–272.966)	0.005	3.481 (1.553–7.800)	0.002

SAS, Self-rating Anxiety Scale; SDS, Self-rating Depression Scale.

^*^Adjusted for age, gender, smoking history, drinking history, BMI, weekly exercise time, pressure score, migraine aura.

In addition, as shown in Supplementary Tables 1,2, further subgroup analyses also revealed significant interactions between anxiety and depression with the risk for poor sleep quality and severe disability degree in age and gender (*p* for interaction <0.05).

### Predictive value of SAS and SDS scores on migraine and the severe burden of migraine

**Figure 3:**
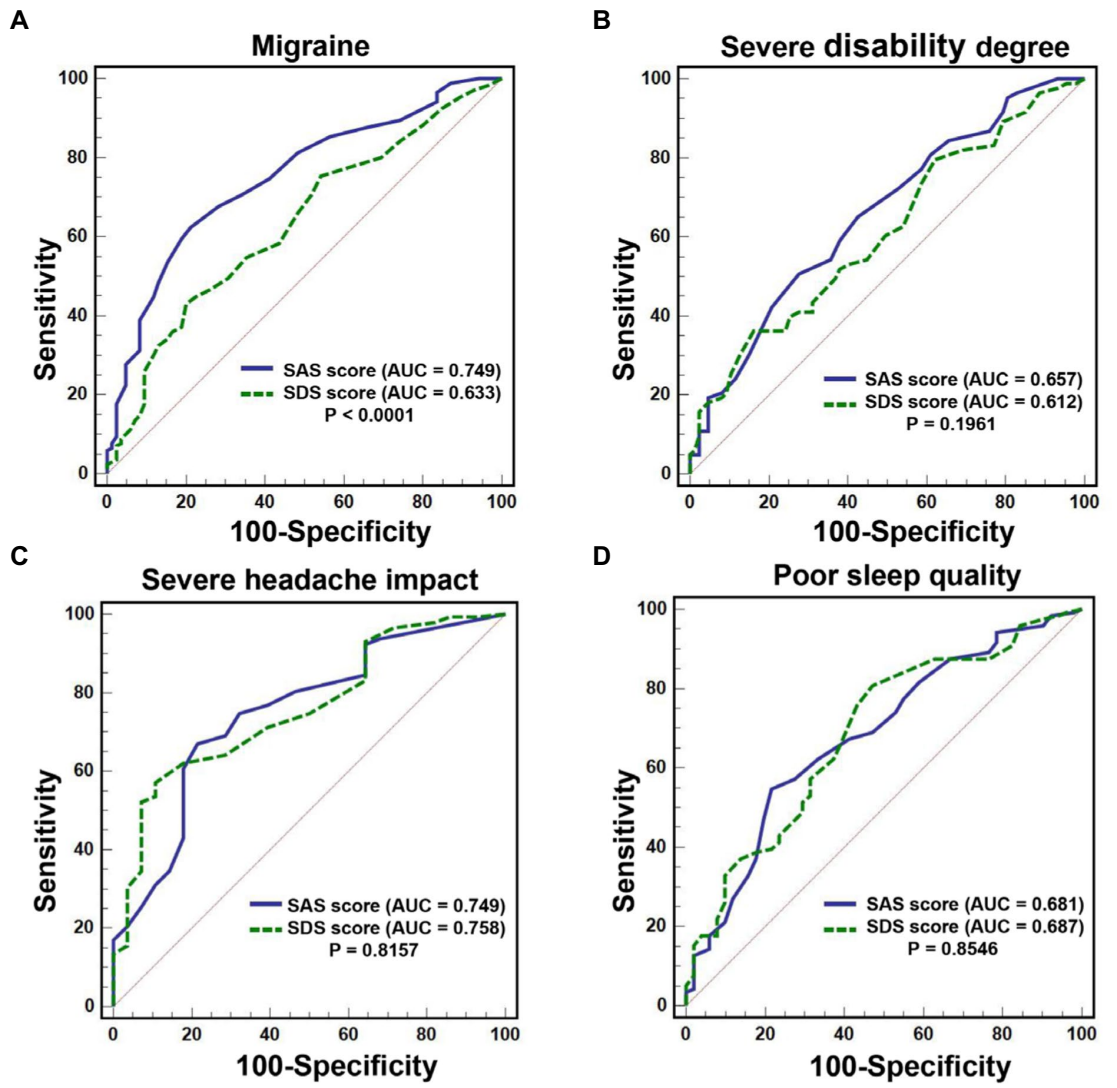
ROC curve of SAS score and SDS score in predicting migraine and the severe burden of migraine. **(A)**: The AUC of SAS score in predicting developing migraine was significantly higher than that of SDS score [0.749 (95%CI: 0.691–0.801) vs. 0.633 (95%CI: 0.571–0.692), *p* < 0.0001]. **(B–D)**: There was no significant difference in the AUC between SAS score and SDS score for predicting poor sleep quality, severe disability degree, and severe headache impact in migraine patients (all *p* > 0.05).

The ROC curves of SAS score and SDS score in predicting migraine and the severe burden of migraine were plotted in Figure 3 and Supplementary Table 3 illustrated the ROC curve results. In addition, the DeLong test was used to compare the area under the ROC curve (AUC) between SAS score and SDS score. As shown in Figure 3A, the AUC of SAS score in predicting developing migraine was significantly higher than that of SDS score in the overall sample [0.749 (95%CI: 0.691–0.801) vs. 0.633 (95%CI: 0.571–0.692), *p* < 0.0001]. However, Figures 3B–D suggested that there was no significant difference in the AUC between SAS score and SDS score for predicting poor sleep quality, severe disability degree, and severe headache impact in migraine patients.

## Discussion

Our previous studies have confirmed a strong relationship between poor sleep quality and migraine and migraine-related burdens (18), but previous sample sizes were too small to allow further statistics on the association of anxiety and depression with migraine and its burdens. Therefore, in the present study, we continued to expand our study sample size and systematically analyzed the associations between anxiety, depression with migraine and migraine-related burdens, aiming to provide new ideas for the prevention and treatment of migraine and its burdens. First, we explored the association of anxiety and depression with the risk of developing migraine through multiple logistic regression analysis in sex-age-matched migraine patients and healthy control subjects, and the results confirmed that both anxiety and depression were significantly positively associated with the increased risk of developing migraine. Meanwhile, the relationship between anxiety and depression with the risk of developing migraine had a significant interaction effect in gender and age. Then, this study illustrated that anxiety and depression were independently associated with the migraine frequency, severity, disability, headache impact, quality of life and sleep quality in migraine patients. Furthermore, anxiety and depression were also significantly positively associated with the risk of poor sleep quality, severe disability degree, and severe headache impact. In addition, this study initially identified the potential predictive value of SAS and SDS scores for migraine and the severe burden of migraine, which may provide new ideas for the prevention and treatment of migraine and its burdens.

In clinical practice, migraine often coexists with a variety of psychiatric disorders. Previous studies have shown that anxiety and depression are the most common psychiatric comorbidities of migraine, affecting the incidence, treatment, and prognosis of migraine (45). Studies have reported that migraineurs have 2–10 times higher rates of depression and anxiety disorders than the general population (46, 47). The present results showed that among 170 migraine patients, 38 had anxiety disorders and 44 had depression, with prevalence rates of 22.4 and 25.9%, respectively, which were 9.5 and 5.5 times higher than those of healthy individuals, similar to those reported in the literature. Multivariate logistic regression analysis showed that after correcting for various confounders, subjects with anxiety and depression still had a significantly increased risk of developing migraine compared to subjects without anxiety and depression, which further confirmed the strong relationship between anxiety and depression and the risk of developing migraine. Considering the differences in migraine prevalence by gender and age, we also further performed a subgroup analysis to explore whether the association of anxiety and depression with developing migraine was influenced by factors such as age and gender. The results showed a significant interaction between anxiety and depression and developing migraine by age and gender, and a stronger association was found in younger and female subjects. The present results may provide a reference for individualized prevention of developing migraine, and we should focus more on anxiety and depression conditions in women and younger populations. For key groups, we may suggest various behavioral interventions, psychological counseling, and drug treatment if necessary to help them alleviate anxiety and depression, and then prevent the occurrence and development of migraine.

The close association of anxiety and depression with migraine may be related to the similar pathogenesis between them. First, the onset of all three is associated with genetic factors. It is generally believed that the pathogenesis of migraine is related to genetic susceptibility and environmental triggers (7). A study showed that both migraine and major depressive disorder are thought to be 50% heritable and are influenced by a large number of genetic variants. Genes implicated in 5-hydroxytryptaminergic and dopaminergic pathways, folate metabolism, GABAergic pathway and growth factor activity may be candidates to confer risk for combined migraine and depression (48). A study found that dopamine D2 receptor genotype was implicated in the pathophysiology of comorbid migraine and depression, as well as reduced tyramine binding, a marker of endogenous depression (49). Tyramine conjugation is reduced in patients with migraine when they have a lifetime history of depression compared to those without migraine. The origin of anxiety may be related to functional and structural alterations in the amygdala. An increased degree of amygdala-anterior cingulate cortex (ACC) connectivity predisposes anxious subjects to focus on attention related to environmental threats (50). Specific risk genotypes for anxiety disorders have also been associated with increased responsiveness of the amygdala and hippocampus to threatening stimuli (51). Recent evidence suggests that both disorders share molecular and cellular mechanisms that control the 5-hydroxytryptamine and glutamatergic neurotransmitter systems. Thus, common genetic factors may underlie the comorbidity of migraine and anxiety disorders. Second, changes in the concentrations of certain neurotransmitters and biochemical factors are common regulatory pathways and mediators of depression, anxiety, and migraine. For example, serotonin dysfunction is an important part of migraine pathogenesis, and it also plays an important role in maintaining and regulating normal mood (52). Dopaminergic dysfunction is a common denominator in the pathophysiology of migraine and depression (53). In addition, several similar brain regions in the ventricles and brainstem and related neurotransmitters are involved in the pathogenesis of depression, anxiety disorders and migraine.

Research on the influencing factors of migraine-related burden is also an important topic of interest. Recent evidences suggest that sleep quality can influence migraine severity (54), and the chronotype seems to influence number and duration of migraine attacks (55). Previous studies have shown that higher migraine frequency is associated with higher symptom scores of anxiety and depression. Results from the Chronic Migraine Epidemiology and Outcomes (CaMEO) study have shown that depression and anxiety are associated with greater headache-related disability after controlling for sociodemographic and headache characteristics (56). In order to more comprehensively explore the relationship between anxiety and depression and migraine-related burdens in migraine patients, not only the migraine frequency and disability degree were investigated in this study, but also the burdens of migraine severity, headache impact, quality of life, and sleep quality were observed. The results showed that after adjusting for confounding factors, anxiety and depression were still significantly associated with migraine frequency, VAS score, MIDAS score, HIT-6 score, MSQ score, and PSQI score, further confirming that the comorbidity of anxiety and depression in migraine patients was strongly associated with increased migraine burden. Further analysis found that both anxiety and depression significantly increased the risk of poor sleep quality, severe headache effects, and severe disability levels in migraineurs. And there were also significant interactions between the association of anxiety and depression with poor sleep quality and severe disability degree in terms of gender and age, which will provide new idea for the prevention and treatment of migraine-related burdens. Interventions targeting depression and anxiety may improve migraine-related burdens in migraineurs.

In addition, considering the significant association of SAS score and SDS score with migraine, this study also preliminarily investigated the predictive value of SAS score and SDS score for migraine and the severe burden of migraine. The results showed that the SAS score had significantly higher predictive accuracy than SDS score and it could be a potential predictor of developing migraine. Although SAS and SDS scores did not differ significantly in predicting the three severe migraine burdens, SDS score still showed good diagnostic specificity for severe headache impact and severe disability degree, with 89.29 and 83.91%, respectively, as well as good predictive sensitivity (80.67%) for poor sleep quality. This preliminary results will provide potentially new ideas for the prevention of severe migraine burden. However, this study was not a prospective study and cannot validate its predictive power, and further validation is needed in the future.

In summary, compared with our previous research, this present study confirmed that anxiety and depression were strongly associated with an increased risk of migraine and found the gender and age differences in their association. Meanwhile, we also found that anxiety and depression were significantly associated with increased burden of headache frequency, severity, disability, quality of life, and sleep quality in migraineurs. And we initially determined the potential predictive value of SAS score and SDS score for migraine and migraine severity burden. The results of this study will provide new ideas for the prevention, diagnosis, and treatment of migraine and its burdens, and have good clinical value. Interventions for depression and anxiety may be an effective way to prevent the development of migraine and improve the migraine-related burden of migraine patients. However, this study also had some limitations. First, anxiety and depression were assessed using SAS scores and SDS scores in this study, although the validity of these methods have been verified in the Chinese population and completed under the guidance of a physician, the scales were difficult to avoid the possible subjectivity. Second, this study used a cross-sectional research method to explore the relationship between anxiety and depression with the migraine burdens in migraine patients, and the causal relationship between them could not be clarified. In addition, the subjects of this study were enrolled from a single center, which may be subject to selection bias. Therefore, the multicenter, large sample size prospective studies are needed in the future to further validate this study.

## Conclusion

Anxiety and depression were significantly independently associated with the increased risk of migraine and migraine-related burdens. Enhanced assessment of SAS score and SDS score is of great clinical value for the early prevention and treatment of migraine and its burdens.

## Data availability statement

The raw data supporting the conclusions of this article will be made available by the authors, without undue reservation.

## Ethics statement

This study was approved by the Ethics Committee of Beijing University of Chinese Medicine (Project No.2022BZYLL0903). Written informed consent for participation was not required for this study in accordance with the national legislation and the institutional requirements.

## Author contributions

SD and ZR: study concept and design and drafting of the manuscript. SD, ZR, HX, ZW, TZ, and GL: acquisition of data. SD, ZR, and HX: analysis and interpretation of data. LL and ZL: revising manuscript for intellectual content. SD and ZL: final approval of the completed manuscript. All authors contributed to the article and approved the submitted version.

## Funding

This study was supported by the Peking University People’s Hospital Talent Introduction Scientific Research Launch Fund (2022-T-02), the China Japan Friendship Hospital Scientific Research Fund (2014-4-QN-33), and the Elite Medical Professionals project of China-Japan Friendship Hospital (no. ZRJY 2021-BJ03).

## Conflict of interest

The authors declare that the research was conducted in the absence of any commercial or financial relationships that could be construed as a potential conflict of interest.

## Publisher’s note

All claims expressed in this article are solely those of the authors and do not necessarily represent those of their affiliated organizations, or those of the publisher, the editors and the reviewers. Any product that may be evaluated in this article, or claim that may be made by its manufacturer, is not guaranteed or endorsed by the publisher.

## Footnotes

^1^ https://www.frontiersin.org/articles/10.3389/fneur.2022.955298/full

## Abbreviations

SAS: Self-rating Anxiety Scale
SDS: Self-rating Depression Scale
PSQI: Pittsburgh Sleep Quality Index
ROC: receiver operating characteristic
AUC: area under the ROC curve
ICHD-III: International Classification of Headache Disorders, 3rd edition
BMI: Body mass index
VAS: Visual analog scale
HIT-6: Headache Impact Test
MIDAS: Migraine Disability Assessment
MSQ: Migraine-Specific Quality-of-Life Questionnaire
