# Supplementary material for: Associations between anxiety, depression with migraine, and migraine-related burdens
Source: Front Neurol. 2023 Apr 25;14:1090878. doi: 10.3389/fneur.2023.1090878 (PMC10166814; doi:10.3389/fneur.2023.1090878)
Supplement: Supplementary file 1 [file Table_1.docx]

**Supplementary Table 1** Subgroup analysis of the effects of anxiety and depression on poor sleep quality in migraine patients.

| Subgroups | Anxiety | | | Depression | | |
| --- | --- | --- | --- | --- | --- | --- |
|  | OR(95%CI) | *P*-value | *P* for interaction^*^ | OR(95%CI) | *P*-value | *P* for interaction^*^ |
| Age |  |  | 0.036 |  |  | 0.005 |
| ≥36 y | 2.401 (0.584-9.872) | 0.225 |  | 3.525(0.878-14.158) | 0.076 |  |
| ＜36 y | 7.103(1.327-38.028) | 0.022 |  | 9.676 (1.701-55.033) | 0.010 |  |
| Gender |  |  | 0.039 |  |  | 0.004 |
| Males | 1.933 (0.010-374.456) | 0.806 |  | 92.22 (0.010-867578.699) | 0.332 |  |
| Females | 2.804 (0.932-8.441) | 0.067 |  | 5.062 (1.624-15.774) | 0.005 |  |

^*^Adjusted for age, gender, smoking history, drinking history, BMI, weekly exercise time, and pressure score, migraine aura.
