# Supplementary material for: Associations between anxiety, depression with migraine, and migraine-related burdens
Source: Front Neurol. 2023 Apr 25;14:1090878. doi: 10.3389/fneur.2023.1090878 (PMC10166814; doi:10.3389/fneur.2023.1090878)
Supplement: Supplementary file 2 [file Table_2.docx]

**Supplementary Table 2** Subgroup analysis of the effects of anxiety and depression on severe disability degree in migraine patients.

| Subgroups | Anxiety | | | Depression | | |
| --- | --- | --- | --- | --- | --- | --- |
|  | OR(95%CI) | *P*-value | *P* for interaction^*^ | OR(95%CI) | *P*-value | *P* for interaction^*^ |
| Age |  |  | 0.011 |  |  | 0.004 |
| ≥36 y | 2.208 (0.679-7.181) | 0.188 |  | 2.463(0.832-7.287) | 0.103 |  |
| ＜36 y | 6.125(1.431-26.217) | 0.015 |  | 5.899 (1.504-23.141) | 0.011 |  |
| Gender |  |  | 0.011 |  |  | 0.002 |
| Males | 15.003 (0.320-704.154) | 0.168 |  | 0.141 (0.004-4.919) | 0.279 |  |
| Females | 3.383 (1.260-9.083) | 0.016 |  | 4.140 (1.684-10.178) | 0.002 |  |

^*^Adjusted for age, gender, smoking history, drinking history, BMI, weekly exercise time, and pressure score,migraine aura.
