# Supplementary material for: Associations between anxiety, depression with migraine, and migraine-related burdens
Source: Front Neurol. 2023 Apr 25;14:1090878. doi: 10.3389/fneur.2023.1090878 (PMC10166814; doi:10.3389/fneur.2023.1090878)
Supplement: Supplementary file 3 [file Table_3.docx]

**Supplementary Table 3** ROC curve results of SAS and SDS scores on migraine and the severe burden of migraine

| Predicted variables | Predictors | AUC (95%CI) | Sensitivity (%) | Specificity (%) | P-value |
| --- | --- | --- | --- | --- | --- |
| Migraine | SAS score | 0.749 (0.691-0.801) | 62.35 | 78.82 | <0.0001 |
|  | SDS score | 0.633 (0.571-0.692) | 42.94 | 80.00 | 0.0003 |
| Poor sleep quality | SAS score | 0.681(0.605-0.750) | 54.62 | 78.43 | 0.0001 |
|  | SDS score | 0.687(0.612-0.756) | 80.67 | 52.94 | <0.0001 |
| Severe headache impact | SAS score | 0.749 (0.677-0.812) | 66.9 | 78.57 | <0.0001 |
|  | SDS score | 0.758(0.686-0.820) | 57.04 | 89.29 | <0.0001 |
| Severe disability degree | SAS score | 0.657 (0.580-0.728) | 50.6 | 72.41 | 0.0002 |
|  | SDS score | 0.612(0.534-0.685) | 36.14 | 83.91 | 0.0096 |

SAS: Self-rating Anxiety Scale; SDS: Self-rating Depression Scale.
